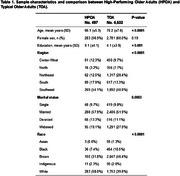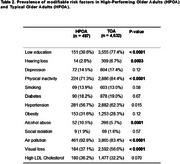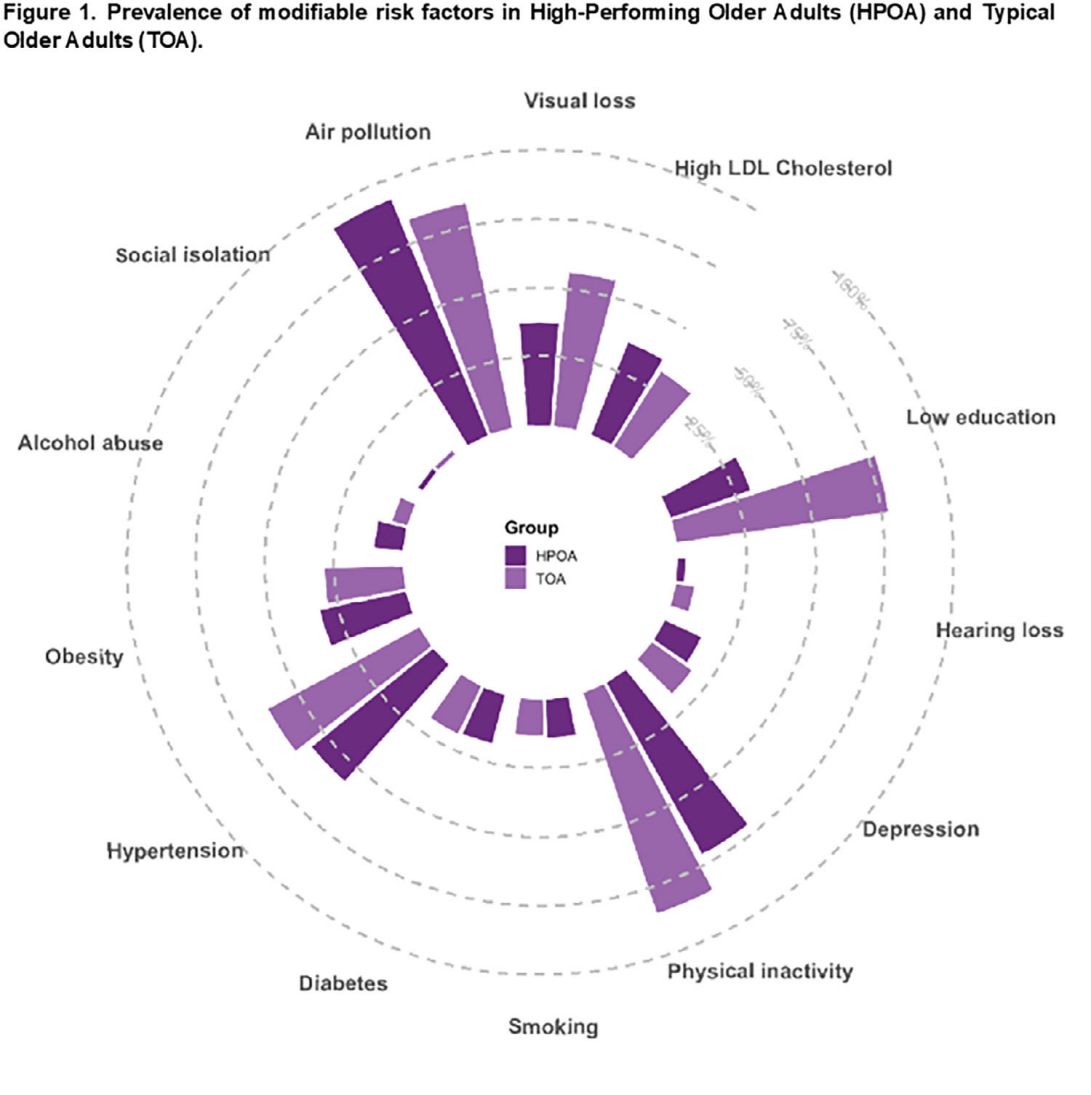# Modifiable risk factors of dementia in Successful Aging: an ELSI‐Brazil study

**DOI:** 10.1002/alz70858_106211

**Published:** 2025-12-26

**Authors:** Rhaná Carolina Santos, Ricardo Nitrini, Adalberto Studart‐Neto, Paulo Caramelli, Eduardo R. Zimmer, Wyllians Vendramini Borelli

**Affiliations:** ^1^ Federal University of Rio Grande do Sul, Porto Alegre, Rio Grande do Sul, Brazil; ^2^ Universidade do Vale do Rio dos Sinos, São Leopoldo, Rio Grande do Sul, Brazil; ^3^ University of São Paulo Medical School, São Paulo, São Paulo, Brazil; ^4^ Cognitive and Behavioral Neurology Unit ‐ University of São Paulo, São Paulo, Brazil; ^5^ Biobank for aging studies of the University of São Paulo Medical School, São Paulo, Brazil; ^6^ Faculdade de Medicina da Universidade de São Paulo, São Paulo, São Paulo, Brazil; ^7^ Behavioral and Cognitive Research Group, Faculdade de Medicina, Universidade Federal de Minas Gerais, Belo Horizonte, Brazil; ^8^ Faculty of Medicine ‐ Universidade Federal de Minas Gerais, Belo Horizonte, Brazil; ^9^ Universidade Federal do Rio Grande do Sul, Porto Alegre, RS, Brazil; ^10^ McGill University, Montreal, QC, Canada; ^11^ Brain Institute of Rio Grande do Sul ‐ Pontifícia Universidade Católica do Rio Grande do Sul, Porto Alegre, Rio Grande do Sul, Brazil; ^12^ Universidade Federal do Rio Grande do Sul, Porto Alegre, Rio Grande do Sul, Brazil; ^13^ Centro de Memória, Hospital Moinhos de Vento, Porto Alegre, RS, Brazil; ^14^ Clinical Hospital of Porto Alegre, Porto Alegre, Rio Grande do Sul, Brazil; ^15^ Brain Institute of Rio Grande do Sul (InsCer), PUCRS, Porto Alegre, Rio Grande do Sul, Brazil

## Abstract

**Background:**

High Performing Older Adults (HPOA) are older individuals with above‐average cognitive abilities, indicating resilience to age‐related pathological processes. Here, we compare HPOA with typical agers, regarding modifiable risk factors of dementia.

**Method:**

Older adults were selected from the ELSI‐Brazil dataset (first wave, 2015‐2016). Cognitive and demographic data was collected for this study. A global cognitive composite z‐score was calculated using orientation (spatial and temporal), memory (immediate and delayed‐recall) and semantic fluency scores. Each score was z‐transformed according to the sample's mean and standard deviation (SD). HPOA was defined as individuals with cognitive z‐scores above 1.5 SD. Typical older adults (TOA) were within ‐1.5 and +1.5 SD from the mean. Thirteen modifiable risk factors were retrieved according to the Lancet Commission: lower levels of education, hearing impairment, high blood pressure, smoking, obesity, depression, high LDL cholesterol, physical inactivity, diabetes, excessive alcohol consumption, air pollution, social isolation, and visual impairment.

**Result:**

Among 9412 individuals from the cohort, 5432 (57.7%) were classified as TOA and 497 were classified as HPOA (Tab. 1). Regarding demographic characteristics, HPOA had a higher educational level (Tab. 1, *p* < 0.001). HPOA showed significantly lower prevalence of lower education, hearing loss, physical inactivity and visual loss (Tab. 2, *p* < 0.001). Contrarily, HPOA had a higher prevalence than typical agers of alcohol abuse and hypertension (Tab. 2, *p* <0.001). There were no significant differences in depression, smoking, diabetes, obesity, and high LDL cholesterol between the groups. Logistic regression models identified that risk factors such as low education (beta = ‐1.8, *p* <0.001), physical inactivity (beta = ‐0.6, *p* < 0.001), and visual loss (beta = ‐0.4, *p* = 0.002) were negatively associated with HPOA. Obesity was positively associated with HPOA (beta = 0.31, *p* = 0.03).

**Conclusion:**

Low education, physical inactivity and visual loss were inversely associated with HPOA. Contrary to expected, the absence of modifiable risk factors of dementia was not proven to be associated with high cognitive abilities. Studying the influence of protective factors in successful aging is essential to underscore their importance in high cognitive performance.